# Norisoprenoids from the Brown Alga *Sargassum naozhouense* Tseng et Lu

**DOI:** 10.3390/molecules23020348

**Published:** 2018-02-07

**Authors:** Yan Peng, Ri-Ming Huang, Xiu-Ping Lin, Yong-Hong Liu

**Affiliations:** 1 Life Science and Technology School, Lingnan Normal University, Cunjin Road 29, Zhanjiang 524048, China; py00_2006@126.com; 2 Key Laboratory of Tropical Marine Bio-Resources and Ecology, Guangdong Key Laboratory of Marine Materia Medica, RNAM Center for Marine Microbiology, South China Sea Institute of Oceanology, Chinese Academy of Sciences, Xinggang West Road 164, Guangzhou 510301, China; xiupinglin@hotmail.com; 3 Guangdong Provincial Key Laboratory of Food Quality and Safety, College of Food Science, South China Agricultural University, Wushan Road 483, Guangzhou 510642, China; huangriming@scau.edu.cn; 4 University of Chinese Academy of Sciences, Yuquan Road 19(A), Beijing 100049, China; 5 South China Sea Bio-Resource Exploitation and Utilization Collaborative Innovation Center, Xinggang West Road 164, Guangzhou 510301, China

**Keywords:** brown alga, *Sargassum naozhouense*, norisoprenoids, cyclopentene

## Abstract

A new C_11_-norisoprenoid derivative, sargassumone (**1**), has been isolated from *Sargassum naozhouense* together with six known norisoprenoids and a highly oxygenated cyclopentene: (2*R*,6*S*,8*S*,9*S*)-hexahydro-2,9-dihydroxy-4,4,8-trimethyl-6-acetyloxy-3(2*H*)-benzofuranone (**2**), (6*S*,8*S*,9*R*)-hexahydro-6,9-dihydroxy-4,4,8-trimethyl-2(2*H*)-benzofuranone (**3**), (6*S*,8*S*,9*R*)-hexahydro-6,9-dihydroxy-4,4,8-trimethyl-2(2*H*)-benzofuranone (**4**), loliolide (**5**), (+)-epiloliolide (**6**), spheciospongones A (**7**), and (+)-kjellmanianone (**8**). Compound **1** was identified on the basis of nuclear magnetic resonance (NMR) and mass spectrometry (MS) analysis, and the absolute stereochemistry was defined by NOESY spectroscopy, minimizing energy calculation, and circular dichroism (CD) spectra. The known compounds **2**–**8**, isolated from *S. naozhouense* for the first time, were identified by comparison of their physical and spectroscopic data with those reported in the literature. Compound **6** was tested for its inhibitory activity against protein tyrosine phosphatase 1B (PTP1B), antioxidant activity with 1,1-diphyl-2-picrylhydrazyl (DPPH) free radicals, and antimicrobial activity against resistant clinical isolates of *Candida albicans*, methicillin-resistant *Staphylococcus aureus (MRSA)*, and *Escherichia coli*.

## 1. Introduction

**Figure 1:**
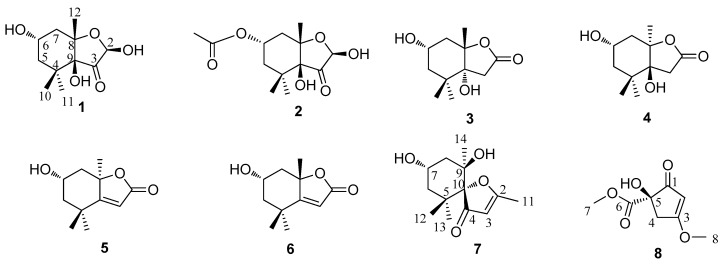
Chemical structures of compounds **1**–**8** isolated from *S. naozhouense*.

*Sargassum naozhouense* Tseng et Lu—one species of the *Sargassum* genus (phylum Phaeophyta, class Cyclosporeae, order Fucales, family Sargassaceae), called “black vegetable”—is distributed widely in the coastal region of the Leizhou Peninsula of China, and can be cultivated commercially under the northern South China Sea’s high-temperature condition [1,2,3,4]. It has a series of pharmaceutical functions in Chinese folk medicine, such as treating infections, laryngitis, dieresis, and other ailments [3]. However, there have been few studies of the chemical constituents that reported primarily carbohydrates, proteins, minerals, dietary fiber, sulfated polysaccharide, and phlorotanns from the brown seaweed *S. naozhouense* [3,5,6]. In order to fully utilize this species and obtain new bioactive compounds from seaweeds being able to produce a great variety of secondary metabolites characterized by a broad spectrum of biological activities, we investigated the brown alga *S. naozhouense*. Previous chemical research on this species has resulted in the isolation of 1-*O*-hexadecanoyl glycerol, pheophytin a, β-sitosterol, mannitol, uracil, thymine, *p*-hydroxybenzoic acid, 3,4-dihydroxybenzoic acid, 4-hydroxyphthalide, and 2′-deoxythmidine [2]. Now, in our further chemical investigation on the EtOH extract of *S. naozhouense*, a new C_11_-norisoprenoid derivative (namely sargassumone (**1**)), and six known norisoprenoids (**2**–**7**), together with a known highly oxygenated cyclopentenone (**8**) (Figure 1) were obtained. Compounds **2**–**8** were also isolated from *S. naozhouense* for the first time. 

In addition, brown algae present anti-diabetic, antioxidant, and antimicrobial effects [7,8,9], and so compound **6** was tested for its inhibitory activity against protein tyrosine phosphatase 1B (PTP1B), used as a potential therapeutic drug target in the treatment of type 2 diabetes, antioxidant activity with 1,1-diphyl-2-picrylhydrazyl (DPPH) free radicals, and antimicrobial activities against resistant clinical isolates of *Candida albicans*, methicillin-resistant *Staphylococcus aureus (MRSA)*, and *Escherichia coli*. 

## 2. Results and Discussion

**Figure 2:**
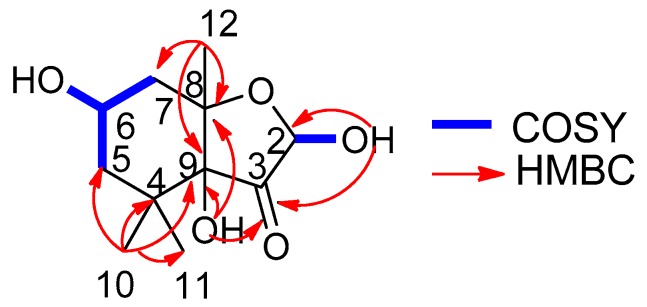
The key HMBC and COSY correlation of compound **1**.

**Figure 3:**
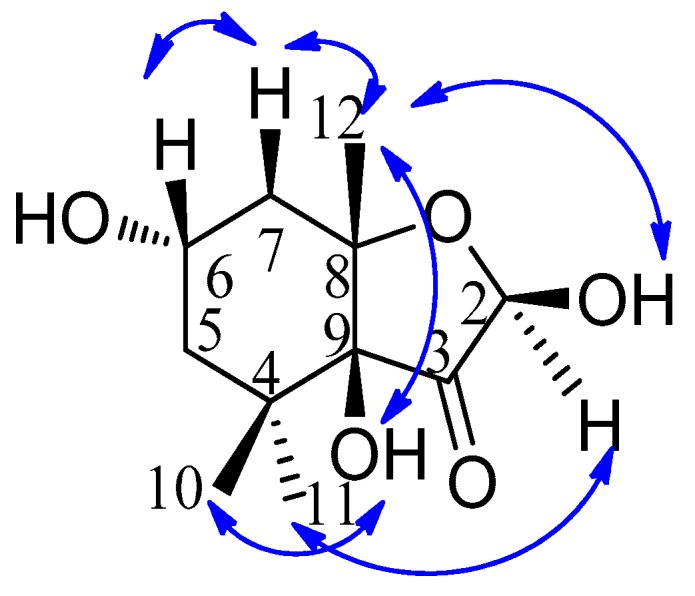
The NOE correlation of compound **1**.

**Figure 4:**
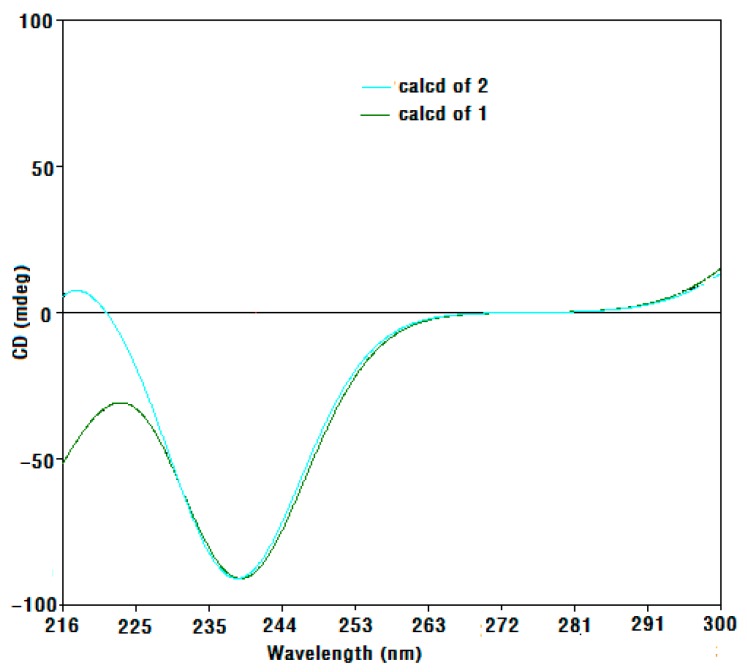
Calculated circular dichroism (CD) spectra of compounds **1** and **2**.

**Figure 5:**
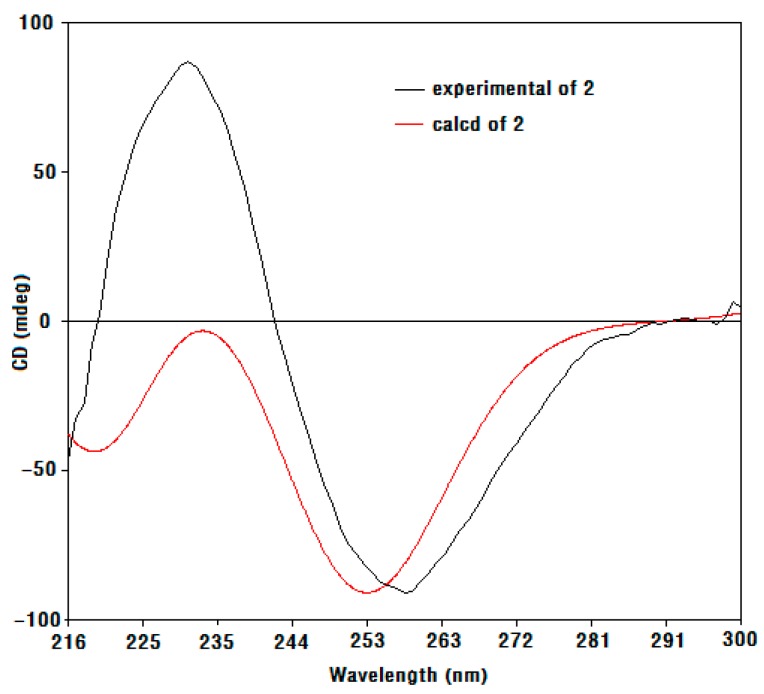
Calculated and experimental CD spectra of compound **2**.

Compound **1** was obtained as a colorless oil, and its molecular formula was determined as C_11_H_18_O_5_ based on electron impact ionization source-mass spectrum (EI-MS) at *m*/*z* 212 [M − H_2_O]^+^ and NMR spectra data (see supplementary materials). The ^1^H-NMR spectra showed the resonances of three methyl singlets at δ 0.86 (3H, s), 0.95 (3H, s), and 1.14 (3H, s), four aliphatic protons at the range of δ 1.27–2.07 attributable to two methylene groups, two hydroxymethine protons at δ 3.79 (1H, m) and 4.93 (1H, d, *J* = 6.0 Hz), and three exchangeable protons at δ 5.36 (9-OH, s), 4.55 (6-OH, d, *J* = 4.5 Hz), and 7.03 (2-OH, d, *J* = 6.0 Hz). The ^13^C-NMR (Table 1), distortionless enchancement by polarization transfer (DEPT), and HMQC spectra revealed the presence of eleven carbons—namely, three methyl carbons, two methylene carbons, two oxymethine carbons, three quaternary carbons (two bearing oxygen), and one ketone carbon, which accounted for the three degrees of unsaturation. The ^1^H-^1^H COSY correlation (Figure 2) from H_2_-5 to H-6 and H-6 to H_2_-7, which established a moiety of CH_2_CHOHCH_2_, in conjunction with the key HMBC correlations from H_3_-10 to C-4, C-5, C-9, and C-11, H_3_-12 to C-7, C-8, and C-9, 9-OH to C-3, C-8, and C-9, and 2-OH to C-2 and C-3 (Figure 2), disclosed the structure of hexahydro-2,6,9-trihydyoxy-4,4,8-trimethyl-3(2*H*)-benzofuranone, which was the deacetyl derivative of compound **2** isolated from the brown alga *Undaria pinnatifida* as a new compound. The NOESY correlations (Figure 3) of H-6/H_a_-7, H_a_-7/H_3_-12, H_3_-12/2-OH and 9-OH, 9-OH/H_3_-10 (δ 0.86, s), and H-2/H_3_-11 (δ 0.95, s) clarified H-6, Ha-7, H_3_-12, 2-OH, 9-OH, and H_3_-10 (δ 0.86, s) being on the same side in opposite to H-2 and H_3_-11 (δ 0.95, s), which were similar to those of compound **2** whose stereochemistry was 2*R*, 6*S*, 8*S*, 9*S* [10], and so the stereochemistry of **1** may be 2*R*, 6*S*, 8*S*, 9*S*. By using a Gaussian-03 package (B3LYP/6-31 G(d) level) for minimizing energy calculation, molecular modeling of **1** suggested its most stable conformation was 2*R*, 6*S*, 8*S*, and 9*S*, which was in agreement with the NOESY results. Furthermore, the calculated circular dichroism (CD) curve of compound **1** was similar to those of compound **2** (Figure 4 and Figure 5), which indicated the same absolute configuration between **1** and **2**. Thus, the structure of **1** was determined to be (2*R*,6*S*,8*S*,9*S*)-hexahydro-2,6,9-trihydroxy-4,4,8-trimethyl-3(2*H*)-benzofuranone, named sargassumon, which is the first example from natural sources to our knowledge.

Compounds **2**–**8** were identified as (2*R*,6*S*,8*S*,9*S*)-hexahydro-2,9-dihydroxy-4,4,8-trimethyl-6-acetyloxy-3(2*H*)-benzofuranone (**2**) [10], (6*S*,8*S*,9*R*)-hexahydro-6,9-dihydroxy-4,4,8-trimethyl-2(2*H*)-benzofuranone (**3**) [10], (6*S*,8*S*,9*R*)-hexahydro-6,9-dihydroxy-4,4,8-trimethyl-2(2*H*)-benzofuranone (**4**) [10], loliolide (**5**) [10,11], (+)-epiloliolide (**6**) [10,11,12], spheciospongones A (**7**) [13], and (+)-kjellmanianone (**8**) [14], respectively, by comparison of their physical and spectroscopic data with those in the literature.

Compound **6** was evaluated for its inhibitory activity as inhibitor of PTP1B in vitro, and the results showed it showed no or weak activity against PTP1B with IC_50_ value of over 50 μmol/L in Table 2. Meanwhile, compound **6** was also evaluated for its antioxidant activity with DPPH free radicals, and the results showed certain antioxidant activity with IC_50_ value of 16.65–17.35 mM in Table 3. In addition, compound **6** was assayed for its antimicrobial activity by the disc diffusion method in vitro. The results of inhibition zones showed that compound **6** exhibited antimicrobial activity against tested resistant strains such as *Candida albicans*, MRSA, and *Escherichia coli*, with inhibition zones ranging from 6.30 to 8.33 mm at a concentration of 4.08 µM in Table 4, which were lower than those of tetracycline used as a positive control at the same concentration. Interestingly, compound **6** exhibited better antimicrobial activities against tested resistant isolates than those of penicillin used as another positive control at the same concentration, which showed no or very weak activity. Furthermore, compound **6** showed antimicrobial activity against the standard strain (ATCC 29213, a non-antibiotic-resistant strain), with the inhibition zone of 7.10–7.90 mm at a concentration of 4.08 µM.

## 3. Materials and Methods

### 3.1. General

Optical rotations were measured with a Perkin-Elmer model 341 polarimeter (Perkin Elmer Corporation, Boston, MA, USA). NMR spectra were recorded on a Bruker Avance 500 NMR spectrometer (Bruker Corporation, Faellanden, Switzerland). HR-ESI-MS spectra were recorded on a Bruker maXis impact mass spectrometer (Bruker Corporation, Bremen, Germany). EI-MS data were measured on a Thermo Scientific DSQ mass spectrometer (Thermo Fisher Scientific Corporation, New York, NY, USA). CD spectra were recorded on a Chirascan CD spectropolarimeter (Applied Photophysics Ltd., Surrey, UK). Column chromatography was performed on silica gel (100–200 and 200–300 mesh, Yantai Jiangyou Silica Gel Development Co., Ltd., Yantai, China) and Sephadex LH-20 (Pharmacia, Uppsala, Sweden). Thin-layer chromatography (TLC) was performed on pre-coated silica gel GF_254_ plates (Yantai Jiangyou Silica Gel Development Co., Ltd., Yantai, China), visualization under UV light, or by heating after spraying with 5% H_2_SO_4_–EtOH (*v*/*v*). Organic solvents for column chromatography were either spectral grade or analytical reagents and obtained from Guangzhou Dongju Test Instrument Co., Ltd. (Guangzhou, China).

Protein tyrosine phosphatase 1B (PTP1B), *p*-nitrophenyl phosphate disodium (*p*NPP), 3-(*N*-morpholino)-propanesulfonic acid (MOPS), and dimethyl sulphoxide (DMSO) were obtained from the National Center for Drug Screening (Shanghai, China). The clinical resistant strains (*Candida albicans 615*, MRSA 315, and *Escherichia coli 234*) were isolated from phlegm samples provided by Guangdong General Hospital (Guangdong, China). The standard strain (*Staphyloccocus aureus ATCC 29213*) was provided by the RNAM Center for Marine Microbiology, South China Sea Institute of Oceanology (Guangzhou, China).

### 3.2. Plant Materials

The brown seaweed *S. naozhouense* was collected in July 2012 from Leizhou Peninsula of Guangdong Province, China. The specimens were identified by Professor Enyi Xie of the College of Fisheries, Guangdong Ocean University. A voucher specimen (No. 20120711) was deposited at the CAS Key Laboratory of Tropical Marine Bio-resources and Ecology, South China Sea Institute of Oceanology, Chinese Academy of Sciences.

### 3.3. Extraction and Isolation

The dried seaweeds (3 kg) were chopped and extracted with 75% EtOH at room temperature for 3 × 7 d. Removal of the solvent in vacuo afforded a syrupy residue (120 g), which was suspended in H_2_O followed by successive partition with petroleum ether, trichloromethane, ethyl acetate, and *n*-butanol. The trichloromethane extract (5.56 g) was subjected to column chromatography (CC) over silica gel (200–300 mesh) eluting with gradient CHCl_3_/acetone (100:0–0:100) to afford four fractions, Frs. 1–4. Fr.1 (100 mg) was subjected to CC eluting with gradient CHCl_3_/MeOH (25:1–10:1) to obtain two subfractions (Frs. 1–1–1–2). Frs. 1–1 was purified by Sephadex LH-20 to yield **1** (1 mg). Frs. 1–2 was directly purified by recrystallization to yield **2** (6 mg). Frs. 2 (200 mg) was further subjected to silica gel column chromatography eluting with CHCl_3_/MeOH (25:1) to yield **3** (20 mg) and **4** (2 mg). Fr. 3 (200 mg) was subjected to CC eluting with CHCl_3_/MeOH (20:1) to yield **5** (1 mg), **6** (50 mg), and **7** (1 mg). Fr. 4 was purified by Sephadex LH-20 to yield **8** (1 mg).

Compound **1**: Colorless oil, [α${]}_{D}^{20}$ +8.4° (*c* 0.018, CHCl_3_). EI-MS (*m*/*z*): 212 [M − H_2_O]^+^, 169, 156, 112, 101, 95, 83, 55; ^1^H-NMR (DMSO-*d*_6_, 500 MHz), see Table 1; ^13^C-NMR (DMSO-*d*_6_, 125 MHz), see Table 1.

Compound **2**: Colorless oil, [α${]}_{D}^{20}$ +16.7° (*c* 0.003, CHCl_3_). CD (CHCl_3_) Δε_258nm_ −0.023, Δε_231nm_ +0.023; ^1^H-NMR (CDCl_3_, 500 MHz) δ: 5.28 (1H, d, *J* = 4.6 Hz, H-2), 5.17 (1H, m, H-6), 3.14 (1H, d, *J* = 4.6 Hz, 2-OH), 2.65 (1H, s, 9-OH), 2.42 (1H, dd, *J* = 14.2, 4.6 Hz, H_a_-7), 1.60 (2H, m, H_b_-7, 5), 1.54 (1H, dd, *J* = 14.2, 11.0 Hz, H_a_-5), 1.31 (3H, s, H-11), 1.15 (3H, s, H-12), 1.01 (3H, s, H-10); ^13^C-NMR (CDCl_3_, 125 MHz) δ: 93.1 (C-2), 214.1 (C-3), 37.8 (C-4), 40.4 (C-5), 66.9 (C-6), 39.2 (C-7), 85.6 (C-8), 81.3 (C-9), 25.6 (C-10), 27.4 (C-11), 25.5 (C-12), 170.5 (C-13), 21.3 (C-14).

Compound **3**: Colorless oil, [α${]}_{D}^{20}$ +8.4° (*c* 0.01, CHCl_3_). ^1^H-NMR (CD_3_OD, 500 MHz) δ: 3.86 (1H, m, H-6), 3.18 (1H, d, *J* = 15.5 Hz, H_a_-3), 2.36 (1H, d, *J* = 17.5 Hz, H_b_-3), 2.31 (1H, dt, *J* = 13.0, 4.0 Hz, H_a_-7), 1.78 (1H, dt, *J* = 13.0,3.0 Hz, H_b_-7), 1.58 (1H, d, *J* = 12.5 Hz, H_a_-5), 1.50 (1H, d, *J* = 12.5 Hz, H_b_-5), 1.56 (3H, s, H-12), 1.08 (3H, s, H-11), 1.00 (3H, s, H-10); ^13^C-NMR (CD_3_OD, 125 MHz) δ: 177.4 (C-2), 42.4 (C-3), 38.1 (C-4), 48.0 (C-5), 64.3 (C-6), 47.3 (C-7), 91.1 (C-8), 82.1 (C-9), 21.3 (C-10), 23.8 (C-11), 27.4 (C-12).

Compound **4**: Colorless oil, [α${]}_{D}^{20}$ −31.9° (*c* 0.0033, CHCl_3_). ^1^H-NMR (DMSO-*d*_6_, 500 MHz) δ: 3.00 (1H, d, *J* = 17.5 Hz, H_a_-3), 2.25 (1H, d, *J* = 17.0 Hz, H_b_-3), 2.00 (1H, dt, *J* = 14.0, 7.0 Hz, H_a_-7), 1.78 (1H, dt, *J* = 14.0, 5.0 Hz, H_b_-7), 1.58 (2H, m, H-5), 1.48 (3H, s, H-12), 1.10 (3H, s, H-11), 0.85 (3H, s, H-10); ^13^C-NMR (DMSO-*d*_6_, 125 MHz) δ: 174.7 (C-2), 40.8 (C-3), 36.8 (C-4), 43.3 (C-5), 63.9 (C-6), 42.7 (C-7), 88.6 (C-8), 79.8 (C-9), 22.5 (C-10), 25.3 (C-11), 27.1 (C-12).

Compound **5**: Colorless needles, [α${]}_{D}^{20}$ −21.3° (*c* 0.003, CHCl_3_). ^1^H-NMR (CDCl_3_, 500 MHz) δ: 5.68 (1H, s, H-3), 4.33 (1H, t, *J* = 3.2 Hz, H-6), 2.47 (1H, d, *J* = 14.0 Hz, H_a_-7), 1.99 (1H, d, *J* = 14.5 Hz, H_a_-5), 1.76 (1H, d, *J* = 3.5 Hz, H_b_-7), 1.54 (1H, dd, *J* = 14.5, 3.5 Hz, H_b_-5), 1.78 (3H, s, H-12), 1.46 (3H, s, H-11), 1.26 (3H, s, H-10); ^13^C-NMR (CDCl_3_, 125 MHz) δ: 182.5 (C-2), 112.9 (C-3), 35.9 (C-4), 47.4 (C-5), 66.8 (C-6), 45.7 (C-7), 86.7 (C-8), 171.6 (C-9), 26.5 (C-10), 27.0 (C-11), 30.7 (C-12).

Compound **6**: Colorless needles, [α${]}_{D}^{20}$ +42.9° (*c* 0.058, CHCl_3_). ^1^H-NMR (CDCl_3_, 500 MHz) δ: 5.65 (1H, s, H-3), 4.10 (1H, m, H-6), 2.50 (1H, ddd, *J* = 12.0, 3.5, 1.5 Hz, H_a_-7), 2.01 (1H, ddd, *J* = 13.0, 4.0, 2.0 Hz, H_a_-5), 1.54(3H, s, H-12), 1.48 (1H, t, *J* = 12.0 Hz, H_b_-7), 1.31(1H, d, *J* = 12.0 Hz, H_b_-5), 1.26 (3H, s, H-11), 1.22 (3H, s, H-10); ^13^C-NMR (CDCl_3_, 125 MHz) δ: 181.4 (C-2), 112.9 (C-3), 35.0 (C-4), 49.6 (C-5), 64.6 (C-6), 47.8 (C-7), 86.8 (C-8), 171.8 (C-9), 25.4 (C-10), 25.0 (C-11), 29.8 (C-12).

Compound **7**: Colorless oil, HR-ESI-MS (*m*/*z*): 263.1799 [M + Na]^+^. CD (MeOH) Δε_265nm_ −1.022; ^1^H-NMR (CDCl_3_, 500 MHz) δ: 5.58 (1H, s, H-3), 4.26 (1H, m, H-7), 2.07 (3H, s, H-11), 2.05 (1H, m, H_a_-8), 1.60~1.68 (2H, m, H-6), 1.51~1.58 (1H, m, H_b_-8), 1.36 (3H, s, H-13), 1.02 (3H, s, H-14), 0.82 (3H, s, H-12); ^13^C-NMR (CDCl_3_, 125 MHz) δ: 190.6 (C-2), 107.3 (C-3), 207.6 (C-4), 23.1 (C-5), 45.9 (C-6), 64.3 (C-7), 44.8 (C-8), 75.5 (C-9), 92.5 (C-10), 16.6 (C-11), 26.3 (C-12), 22.2 (C-13), 25.8 (C-14).

Compound **8**: Colorless crystals, [α${]}_{D}^{20}$ +1.6° (*c* 1.80, CHCl_3_). ^1^H-NMR (CDCl_3_, 500 MHz) δ: 5.34 (1H, s, H-2), 3.94 (3H, s, H-7), 3.80 (3H, s, H-8), 3.20 (1H, d, *J* = 17.5 Hz, H_a_-4), 2.76 (1H, d, *J* = 17.5 Hz, H_b_-4).

### 3.4. Bioactivity Assay

#### 3.4.1. PTP1B Inhibition Assay

PTP1B (protein tyrosine phosphatase 1B) was expressed and purified in *E. coli*, and the enzymatic activities were measured at 30 °C by monitoring the hydrolysis of *p*NPP [15]. The enzymatic activities of PTP1B catalytic domain were also determined at 30 °C by monitoring the hydrolysis of *p*NPP. Dephosphorylation of *p*NPP generates *p*-nitrophenol, which can be monitored spectrophotometrically at 405 nm. In a 100-μL assay mixture containing 50 mM MOPS, pH 6.5, 2 mM *p*NPP, and recombinant enzymes, PTP1B activities were continuously monitored and the initial rate of the hydrolysis was determined from the early linear region of the enzymatic reaction kinetic curve as described by Zhang et al. [15].

The inhibitory activity of Compound **6** was tested against PTP1B by the colorimetric assay described by Zhang et al. Briefly, the compound was solubilized in DMSO at 20 μg/mL, and then added to a 96-well clear polystyrene plate (Corning, Action, MA, USA) with oleanolic acid (12.5 mM) as the positive inhibition. After adding an assay mixture (88 μL), 10 μL 300 nM GST-PTP1B was added to initiate the reaction. Then, the catalysis of *p*NPP was continuously monitored on SpectraMax 340 microplate reader (Molecular Devices, Sunnyvale, CA, USA) at 405 nm for 2 min at 30 °C in a final 100 μL volume containing 50 mM MOPS, pH 6.5, 2 mM *p*NPP, 30 nM PTP1B, and 2% DMSO. The IC_50_ value was calculated from the non-linear curve fitting percent inhibition (% inhibition) to inhibitor concentration [*I*] by the following equation, where k is the Hill coefficient:

% Inhibition = 100/{(1 + (IC_50_)/[*I*])k}. (1)

#### 3.4.2. DPPH Free Radical Scavenging Assay

The free radical scavenging potential of compound **6** was evaluated using the DPPH free radical scavenging assay described by Sharma and Bhat [16]. A total of 200 μL of the reaction mixture in a 96-well plate was composed of 100 μL of 0.1 mM DPPH (Sigma Aldrich, St. Louis, MO, USA) in methanol and 100 μL of different concentrations of test compound. The reaction mixture was then shaken well and incubated for 30 min in darkness at 37 °C. Meanwhile, a sample composed of 100 μL of methanol with 100 μL of 0.1 mM DPPH was taken as a control, while vitamin C (Shanxi Yishengtang Pharmaceutical Co., Ltd., Tongchuan, China) was taken as a reference material. The absorbance of reaction solution was measured at 517 nm with a plate reader (Thermo Multiskan MK3 spectrophotometer, Thermo Fisher Scientific, Waltham, MA, USA), and the percentage of scavenging capacity (%) was calculated by the following equation, where A_Control_ stands for the absorbance of the control and A_Treatment_ stands the absorbance of the treatment:

Scavenging capacity (%) = (1 − A_Treatment_/A_Control_) × 100. (2)

The DPPH IC_50_ value is the concentration required to scavenge DPPH radical by 50%.

#### 3.4.3. Antimicrobial Activity

Antimicrobial test for compound **6** was carried out against resistant microbes such as *Candida albicans*, MRSA, and *Escherichia coli* by disc diffusion method [17]. Briefly, a Petri dish was prepared with a base layer of Muller Hinton (MH) agar (10 mL), and then 0.1 mL culture with the concentration of 10^5^ CFU/mL was uniformly distributed on to MH agar plates. Paper discs with the diameter of 5 mm, made and sterilized, were placed on the surface of MH agar plates at a distance of 2 cm. Drugs with the concentration of 800 μg/mL were added on each disc and incubated at 37 °C or 20 °C for 8–10 h. After incubation, the diameters of the inhibition zones were measured.

### 3.5. Statistical Analysis

For the bioactivity assay, all determinations were triplicates, and data were expressed as mean and standard deviation (SD). Analysis of variance was performed, and the mean separation was done by the least significant difference (LSD) (*p* ≤ 0.05) using SPSS 18.0 software for Windows (SPSS Inc., Chicago, IL, USA). 

## 4. Conclusions

The phytochemical investigation of *S. naozhouense* afforded a new C_11_-norisoprenoid derivative and six known norisoprenoids, as well as a known cyclopentenone derivative. All isolates were isolated from *S. naozhouense* for the first time. Compound **6** showed a marginal inhibitory activity against PTP1B, certain antioxidant activity against DPPH radicals, and antimicrobial activities against some resistant microbes such as *Candida albicans*, MRSA, and *Escherichia coli*.

## Figures and Tables

**Table 1:** ^1^H- (500 MHz) and ^13^C-NMR (125 MHz) data of compound **1** (in DMSO-*d*_6_, δ in ppm, *J* in Hz).

Position	δ_C_	δ_H_	HMBC (H→C)
2	92.6	4.93 d (6.0)	84.6
3	214.8		
4	37.1		
5	44.4	1.36 m	
6	61.6	3.79 m	
7	43.1	2.07 dd (4.5, 4.5)	44.4, 61.6, 80.6, 84.6
8	84.6		
9	80.6		
10	26.1	0.86 s	25.7, 37.1, 44.4, 80.6
11	25.7	0.95 s	26.1, 37.1, 44.4, 80.6
12	27.6	1.14 s	43.1, 80.6, 84.6
2-OH		7.03 d (6.0)	92.6, 214.8
6-OH		4.55 d (4.5)	44.4
9-OH		5.36 s	37.1, 80.6, 84.6, 214.8

**Table 2:** Protein tyrosine phosphatase 1B (PTP1B) inhibitory activity of compound **6**.

Compounds	Inhibitory Activity (IC_50_, μmol/L) ^#^
**6**	>50
Oleanolic acid	1.9 ± 0.3

^#^ IC_50_ values were determined by regression analysis and expressed as mean ± SD of three replicates.

**Table 3:** 1,1-Diphyl-2-picrylhydrazyl (DPPH) free radical scavenging activity of compound **6** ^#^.

Compounds	IC_50_ (mM)
**6**	17 ± 0.35
Vitamin C	0.11 ± 0.01

^#^ mean ± SD of three replicates.

**Table 4:** Antimicrobial activity of compound **6**.

Strains	Zone of Inhibition (mm) ^#^		
	6	Tetracycline	Penicillin
*Candida albicans 615*	6.50 ± 0.20	10.50 ± 0.23	–
MRSA 315	8.20 ± 0.11	11.00 ± 0.10	–
*Escherichia coli 234*	7.00 ± 0.20	12.00 ± 0.06	–
*Staphyloccocus aureus 29213*	7.50 ± 0.40	9.50 ± 0.52	11.00 ± 0.05

^#^: mean ± SD of three replicates; –: no or very weak activity. MRSA: methicillin-resistant *Staphylococcus aureus*.

## Supplementary Materials

The following are available online.

## References

[1] Huang Z.G. (1994). Marine Species and Their Distributions in China’s Seas.

[2] Peng Y., Gao W.H., Lin X.P., Yan T., Liu Y.H. (2014). Chemical constituents of seaweed *Sargassum naozhouense*. J. Chin. Med. Mater..

[3] Peng Y., Xie E., Zheng K., Fredimoses M., Yang X., Zhou X., Wang Y., Yang B., Lin X., Liu J. (2013). Nutritional and chemical composition and antiviral activity of cultivated seaweed *Sargassum naozhouense* Tseng et Lu. Mar. Drugs.

[4] Xie E.Y., Liu B.C., Jia C., Chen X.L., Yang B. (2013). Artificial seed production and cultivation of the edible brown alga *Sargassum naozhouense* Tseng et Lu. J. Appl. Phycol..

[5] Lu H., Chen X., Ou X., Mao J., Ji H. (2013). Anticoagulant activity of phlorotannins from *S. naozhouense*. Nat. Prod. Res. Dev..

[6] Feng M., Li Y., Cui L., Liu Y. (2015). The protective effect of phlorotannins from *Sargassum naozhouense* on bone loss in ovariectomized mice with hyperlipidemia. Chin. J. Osteoporos..

[7] Sharifuddin Y., Chin Y.X., Lim P.E., Phang S.M. (2015). Potential Bioactive compounds from seaweed for diabetes management. Mar. Drugs.

[8] Heo S.J., Park E.J., Lee K.W., Jeon Y.J. (2005). Antioxidant activities of enzymatic extracts from brown seaweeds. Bioresour. Technol..

[9] Shanmughapriya S., Manilal A., Sujith S., Selvin J., Seghal Kiran G., Natarajaseenivasan K. (2008). Antimicrobial activity of seaweeds extracts against multiresistant pathogens. Ann. Microbiol..

[10] Kimura J., Maki N. (2002). New loliolide derivatives from the brown alga *Undaria pinnatifida*. J. Nat. Prod..

[11] Chávez J.P., Santos I.D.D., Cruz F.G., David J.M., Yang S.W., Cordell G.A. (1997). A quinoline alkaloid from *Acanthosyris Paulo-alvinii*. Phytochemistry.

[12] Park K.E., Kim Y.A., Jung H.A., Lee H.J., Ahn J.W., Lee B.J., Seo Y.W. (2004). Three norisoprenoids from the brown alga *Sargassum thunbergii*. J. Korean Chem. Soc..

[13] Liu D., Xu M.J., Wu L.J., Deng Z.W., Lin W.H. (2009). Norisoprenoids from the marine sponge *Spheciospongia* sp.. J. Asian Nat. Prod. Res..

[14] Nakayama M., Fukuoka Y., Nozaki H., Matsuo A., Hayashi S. (1980). Structure of (+)-kjellmanianone, a highly oxygenated cyclopentenone from the marin alga, *Sargassum kjellmanianum*. Chem. Lett..

[15] Zhang W., Hong D., Zhou Y.Y., Zhang Y.N., Shen Q., Li J.Y., Hu L.H., Li J. (2006). Ursolic acid and its derivative inhibit protein tyrosine phosphatase 1B, enhancing insulin receptor phosphorylation and stimulation glucose uptake. Biochim. Biophys. Acta.

[16] Sharma O.P., Bhat T.K. (2009). DPPH antioxidant assay revisited. Food Chem..

[17] Santhosh S.K., Venugopal A., Radhakrishnan M.C. (2016). Study on the phytochemical, antibacterial and antioxidant activities of *Simarouba glauca*. Indian J. Biol. Sci..

