# Supplementary material for: Norisoprenoids from the Brown Alga Sargassum naozhouense Tseng et Lu
Source: Molecules. 2018 Feb 7;23(2):348. doi: 10.3390/molecules23020348 (PMC6017521; doi:10.3390/molecules23020348)
Supplement: Supplementary file 1 [file molecules-23-00348-s001.zip › Supplementary files/2(H╞╫).pdf]

H NMR Spectrum of S-E-3(1)

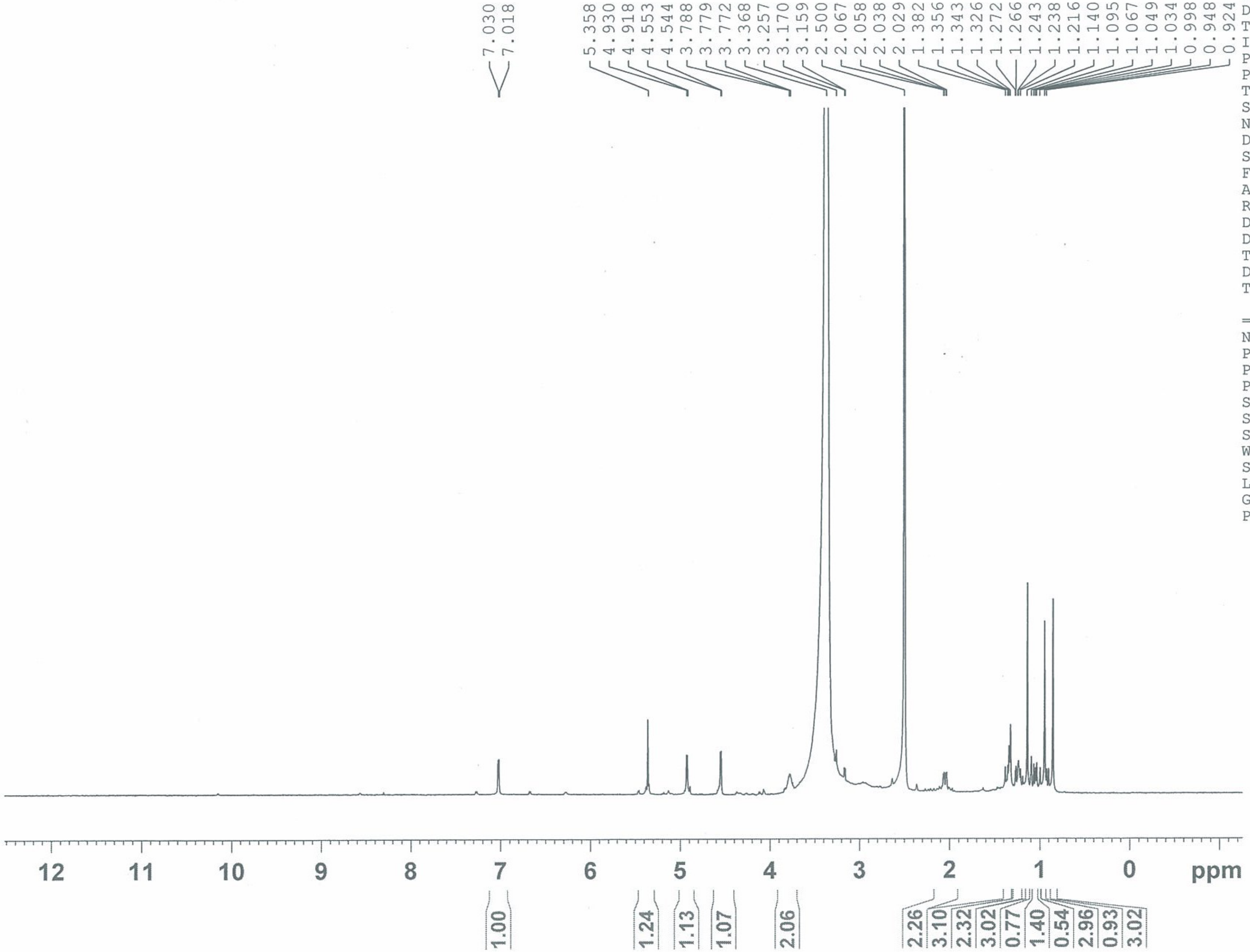

NAME	pengyan-S-E-3(1)
EXPNO	1
PROCNO	1
Date_	20120827
Time	11.49
INSTRUM	spect
PROBHD	5 mm PABBO BB-
PULPROG	zg30
TD	65536
SOLVENT	DMSO
NS	66
DS	2
SWH	10330.578 Hz
FIDRES	0.157632 Hz
AQ	3.1720407 se
RG	128
DW	48.400 us
DE	6.50 us
TE	297.9 K
D1	1.00000000 se
TD0	1

======== CHANNEL f1 ======

NUC1	1H
P1	13.50 us
PL1	1.00 dB
PL1W	8.77915382 W
SFO1	500.1330885 MH
SI	32768
SF	500.1300052 MH
WDW	EM
SSB	0
LB	0.30 Hz
GB	0
PC	1.00
