# Supplementary material for: Norisoprenoids from the Brown Alga Sargassum naozhouense Tseng et Lu
Source: Molecules. 2018 Feb 7;23(2):348. doi: 10.3390/molecules23020348 (PMC6017521; doi:10.3390/molecules23020348)
Supplement: Supplementary file 1 [file molecules-23-00348-s001.zip › Supplementary files/4(C╞╫2).pdf]

13C NMR Spectrum of S-E-3(1)

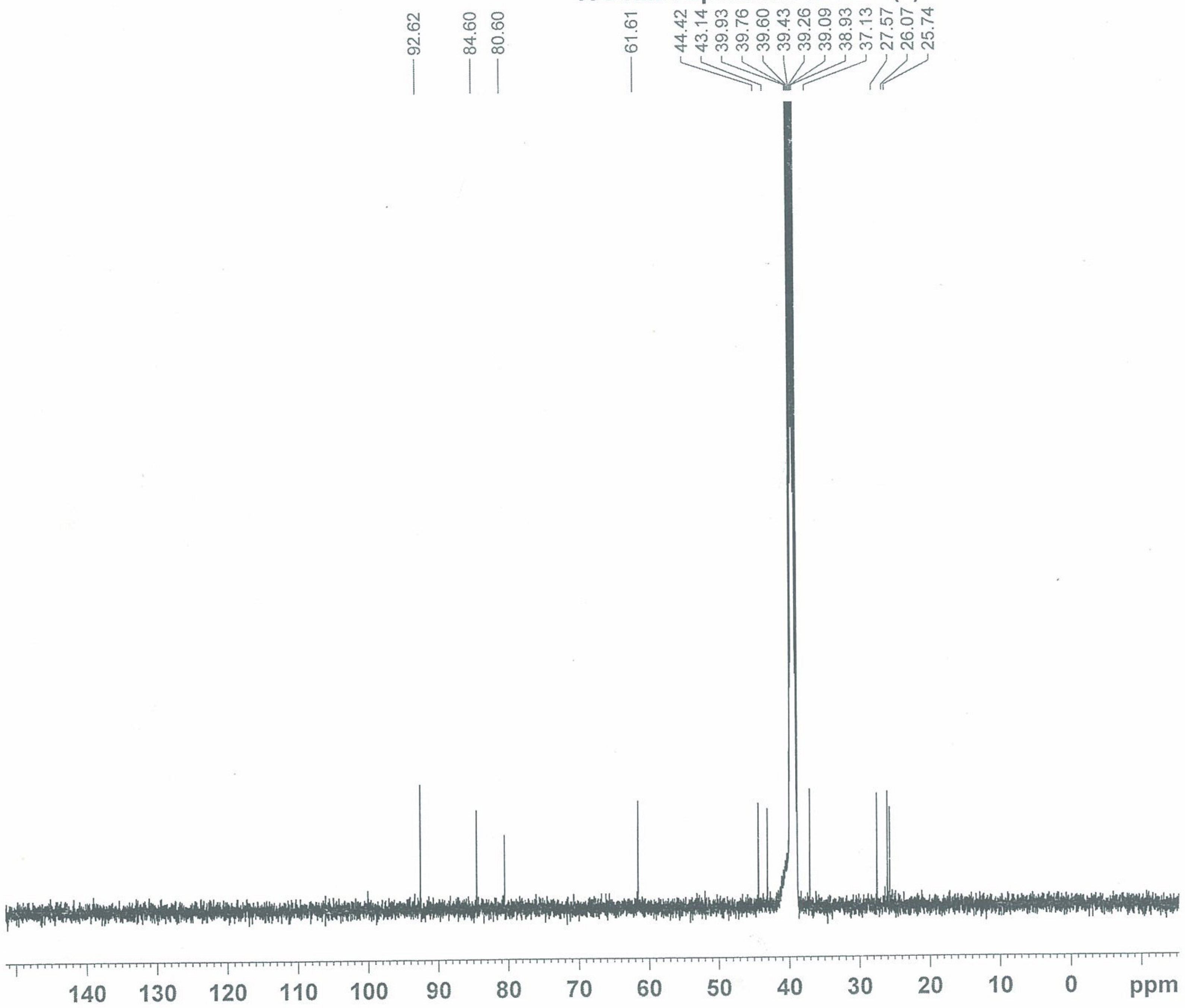

NAME	pengyan-S-E-3(1)	
EXPNO	2	
PROCNO	1	
Date_	20120906	
Time	20.31	
INSTRUM	spect	
PROBHD	5 mm PABBO BB-	
PULPROG	zgpg30	
TD	32768	
SOLVENT	DMSO	
NS	10240	
DS	4	
SWH	30030.029	Hz
FIDRES	0.916444	Hz
AQ	0.5456539	sec
RG	9195.2	
DW	16.650	usec
DE	6.50	usec
TE	298.8	K
D1	1.00000000	sec
D11	0.03000000	sec
TD0	1	

======== CHANNEL f1 ========

NUC1	13C	
P1	10.00	usec
PL1	0.00	dB
PL1W	100.47545624	W
SFO1	125.7703643	MHz

======== CHANNEL f2 ========

CPDPRG2	waltz16	
NUC2	1H	
PCPD2	80.00	usec
PL2	1.00	dB
PL12	16.46	dB
PL13	17.46	dB
PL2W	8.77915382	W
PL12W	0.24971968	W
PL13W	0.19835939	W
SFO2	500.1320005	MHz
SI	32768	
SF	125.7578658	MHz
WDW	EM	
SSB	0	
LB	1.00	Hz
GB	0	
PC	1.40	
