# Supplementary material for: Norisoprenoids from the Brown Alga Sargassum naozhouense Tseng et Lu
Source: Molecules. 2018 Feb 7;23(2):348. doi: 10.3390/molecules23020348 (PMC6017521; doi:10.3390/molecules23020348)
Supplement: Supplementary file 1 [file molecules-23-00348-s001.zip › Supplementary files/6(HSQC╞╫).pdf]

HSQC NMR Spectrum of S-E-3(1)

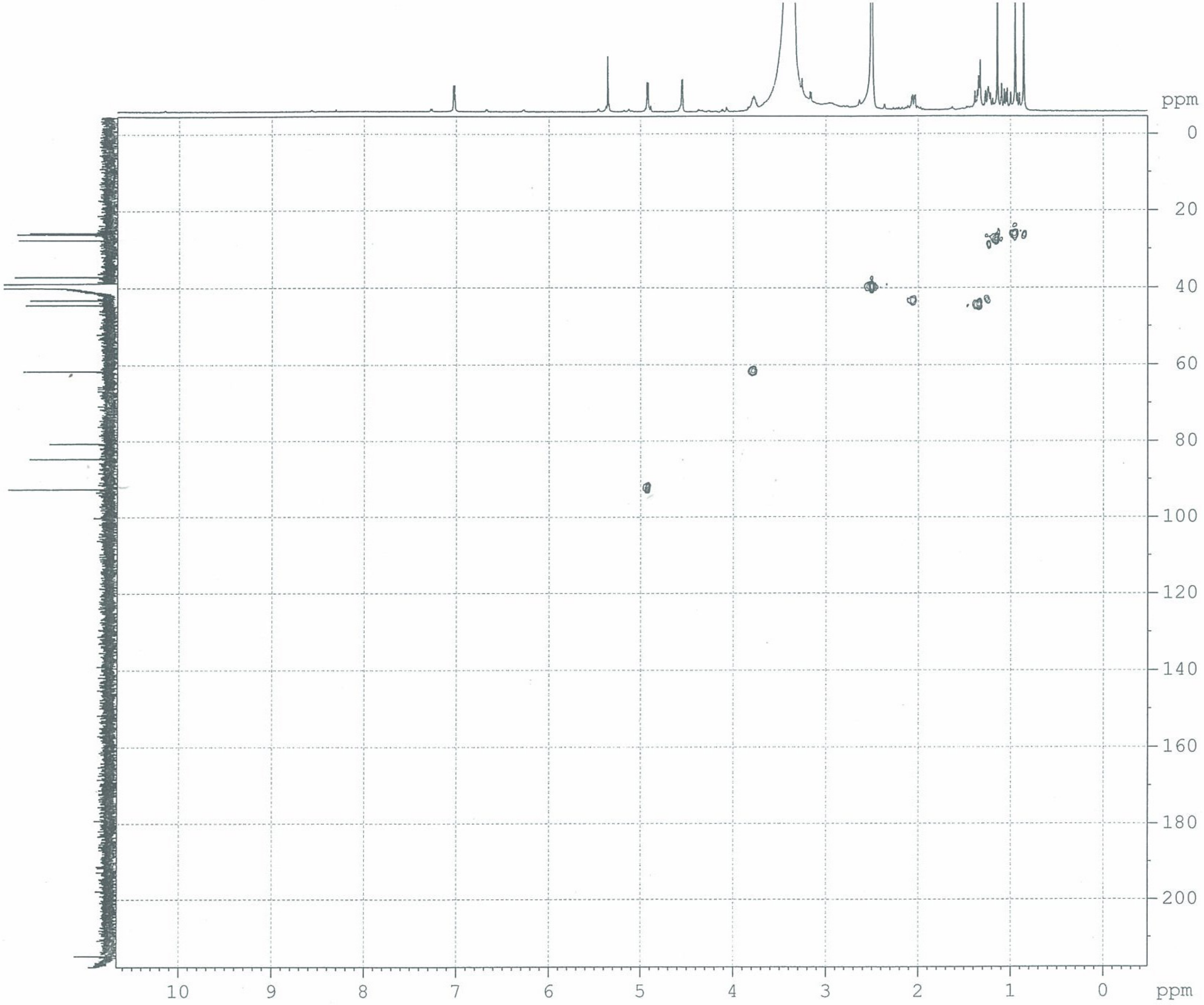

NAME	pengyan-S-E-3(1)
EXPNO	5
PROCNO	1
Date_	20120907
Time	23.34
INSTRUM	spect
PROBHD	5 mm PABBO BB-
PULPROG	hsqcedetgp
TD	1024
SOLVENT	DMSO
NS	32
DS	16
SWH	5580.357 Hz
FIDRES	5.449567 Hz
AQ	0.0918900 sec
RG	18390.4
DW	89.600 usec
DE	6.50 usec
TE	298.0 K
CNST2	145.0000000
D0	0.00000300 sec
D1	1.00000000 sec
D4	0.00172414 sec
D11	0.03000000 sec
D13	0.00000400 sec
D16	0.00020000 sec
D21	0.00345000 sec
IN0	0.00001785 sec
ZGOPTNS	
======== CHANNEL f1 ========	
NUC1	1H
P1	13.50 usec
P2	27.00 usec
P28	0.00 usec
PL1	1.00 dB
PL1W	8.77915382 W
SFO1	500.1325507 MHz
======== CHANNEL f2 ========	
CPDPRG2	garp
NUC2	13C
P3	10.00 usec
P4	20.00 usec
PCPD2	65.00 usec
PL2	0.00 dB
PL12	16.26 dB
PL2W	100.47545624 W
PL12W	2.37716842 W
SFO2	125.7712577 MHz
====== GRADIENT CHANNEL ====	
GPNAM1	SINE.100
GPNAM2	SINE.100
GPZ1	80.00 %
GPZ2	20.10 %
P16	1000.00 usec
ND0	2
TD	128
SFO1	125.7713 MHz
FIDRES	218.822342 Hz
SW	222.700 ppm
FnMODE	Echo-Antiecho
SI	1024
SF	500.1300052 MHz
[illegible]	[illegible]
