# Supplementary material for: Norisoprenoids from the Brown Alga Sargassum naozhouense Tseng et Lu
Source: Molecules. 2018 Feb 7;23(2):348. doi: 10.3390/molecules23020348 (PMC6017521; doi:10.3390/molecules23020348)
Supplement: Supplementary file 1 [file molecules-23-00348-s001.zip › Supplementary files/7(COSY╞╫).pdf]

COSY NMR Spectrum of S-E-3(1)

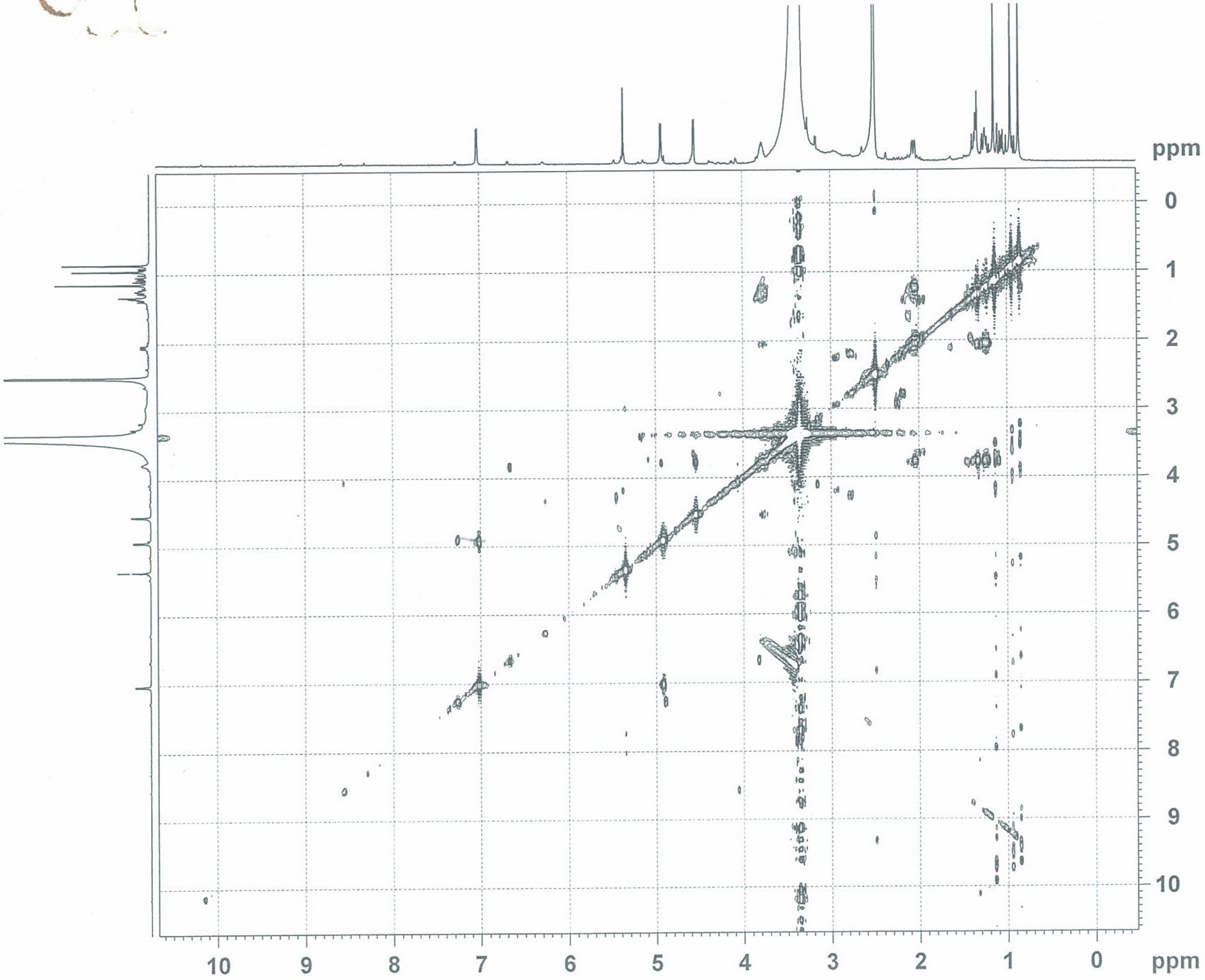

NAME	pengyan-S-E-3(1)
EXPNO	6
PROCNO	1
Date_	20120907
Time	20.47
INSTRUM	spect
PROBHD	5 mm PABBO BB-
PULPROG	cosygpqf
TD	2048
SOLVENT	DMSO
NS	24
DS	8
SWH	5580.357 Hz
FIDRES	2.724784 Hz
AQ	0.1836404 sec
RG	64
DW	89.600 usec
DE	6.50 usec
TE	297.9 K
D0	0.00000300 sec
D1	1.00000000 sec
D13	0.00000400 sec
D16	0.00020000 sec
IN0	0.00017920 sec
======== CHANNEL f1 ========	
NUC1	1H
P0	13.50 usec
P1	13.50 usec
PL1	1.00 dB
PL1W	8.77915382 W
SFO1	500.1325507 MHz
====== GRADIENT CHANNEL =====	
GPNAM1	SINE.100
GPZ1	10.00 %
P16	1000.00 usec
ND0	1
TD	128
SFO1	500.1326 MHz
FIDRES	43.596321 Hz
SW	11.158 ppm
FnMODE	QF
SI	1024
SF	500.1300052 MHz
WDW	SINE
SSB	0
LB	0.00 Hz
GB	0
PC	1.40
SI	1024
MC2	QF
SF	500.1300052 MHz
WDW	SINE
SSB	0
LB	0.00 Hz
GB	0
