# Supplementary material for: Norisoprenoids from the Brown Alga Sargassum naozhouense Tseng et Lu
Source: Molecules. 2018 Feb 7;23(2):348. doi: 10.3390/molecules23020348 (PMC6017521; doi:10.3390/molecules23020348)
Supplement: Supplementary file 1 [file molecules-23-00348-s001.zip › Supplementary files/8(HMBC╞╫).pdf]

HMBC NMR Spectrum of S-E-3(1)

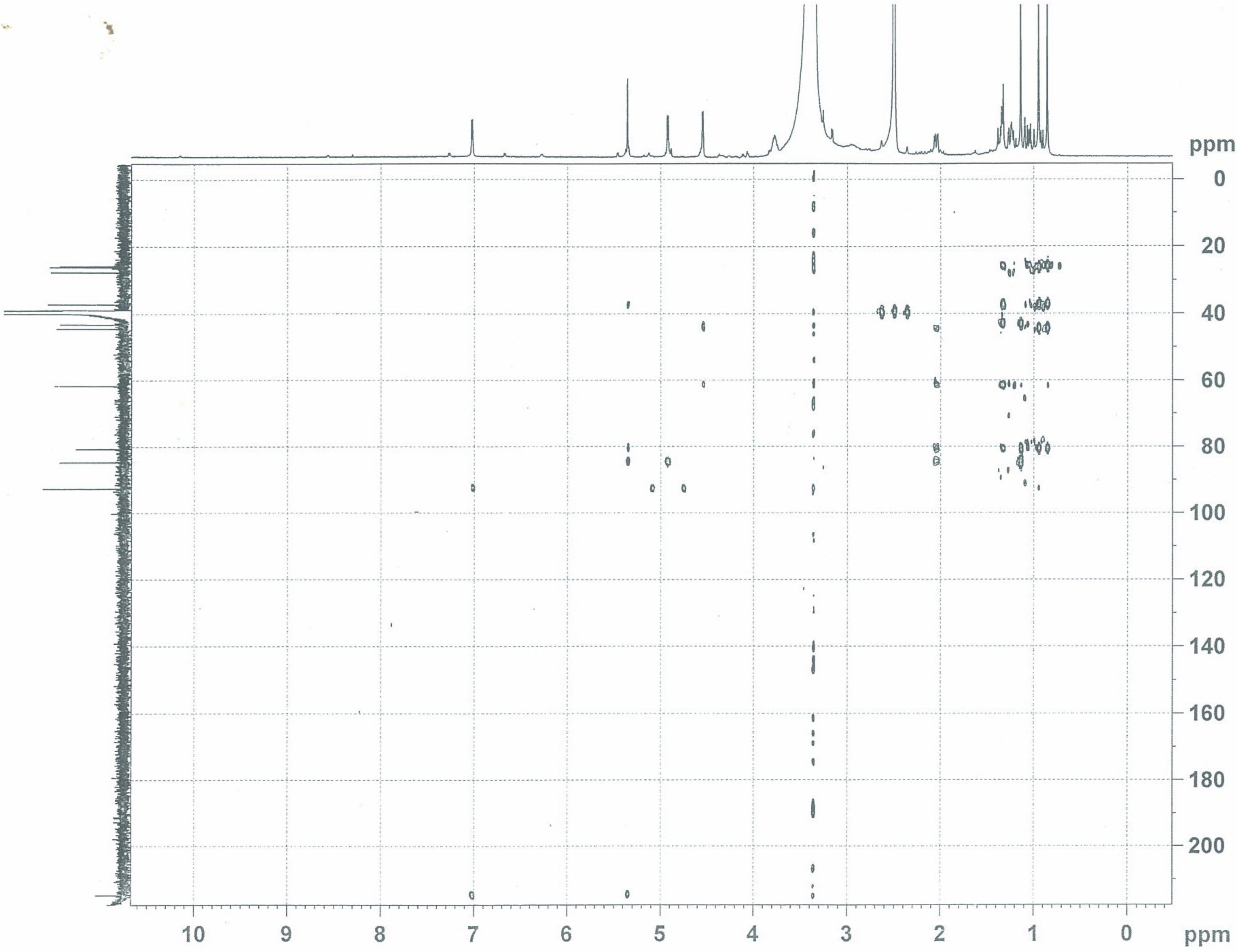

NAME	pengyan-S-E-3(1)	
EXPNO	4	
PROCNO	1	
Date_	20120907	
Time	17.55	
INSTRUM	spect	
PROBHD	5 mm PABBO BB-	
PULPROG	hmbcgpndqf	
TD	4096	
SOLVENT	DMSO	
NS	56	
DS	16	
SWH	5580.357	Hz
FIDRES	1.362392	Hz
AQ	0.3671412	sec
RG	16384	
DW	89.600	usec
DE	6.50	usec
TE	298.4	K
CNST13	8.0000000	
D0	0.00000300	sec
D1	1.00000000	sec
D6	0.06250000	sec
D16	0.00020000	sec
IN0	0.00001785	sec
======== CHANNEL f1 ========		
NUC1	1H	
P1	13.50	usec
P2	27.00	usec
PL1	1.00	dB
PL1W	8.77915382	W
SFO1	500.1325507	MHz
======== CHANNEL f2 ========		
NUC2	13C	
P3	10.00	usec
PL2	0.00	dB
PL2W	100.47545624	W
SFO2	125.7712577	MHz
====== GRADIENT CHANNEL =====		
GPNAM1	SINE.100	
GPNAM2	SINE.100	
GPNAM3	SINE.100	
GPZ1	50.00	%
GPZ2	30.00	%
GPZ3	40.10	%
P16	1000.00	usec
ND0	2	
TD	128	
SFO1	125.7713	MHz
FIDRES	218.822342	Hz
SW	222.700	ppm
FnMODE	QF	
SI	1024	
SF	500.1300052	MHz
WDW	SINE	
SSB	0	
LB	0.00	Hz
GB	0	
PC	1.40	
SI	1024	
MC2	QF	
SF	125.7578658	MHz
WDW	SINE	
SSB	0	
LB	0.00	Hz
GB	0	
