# Supplementary material for: Norisoprenoids from the Brown Alga Sargassum naozhouense Tseng et Lu
Source: Molecules. 2018 Feb 7;23(2):348. doi: 10.3390/molecules23020348 (PMC6017521; doi:10.3390/molecules23020348)
Supplement: Supplementary file 1 [file molecules-23-00348-s001.zip › Supplementary files/9(NOE╞╫).pdf]

NOE NMR Spectrum of S-E-3(1)

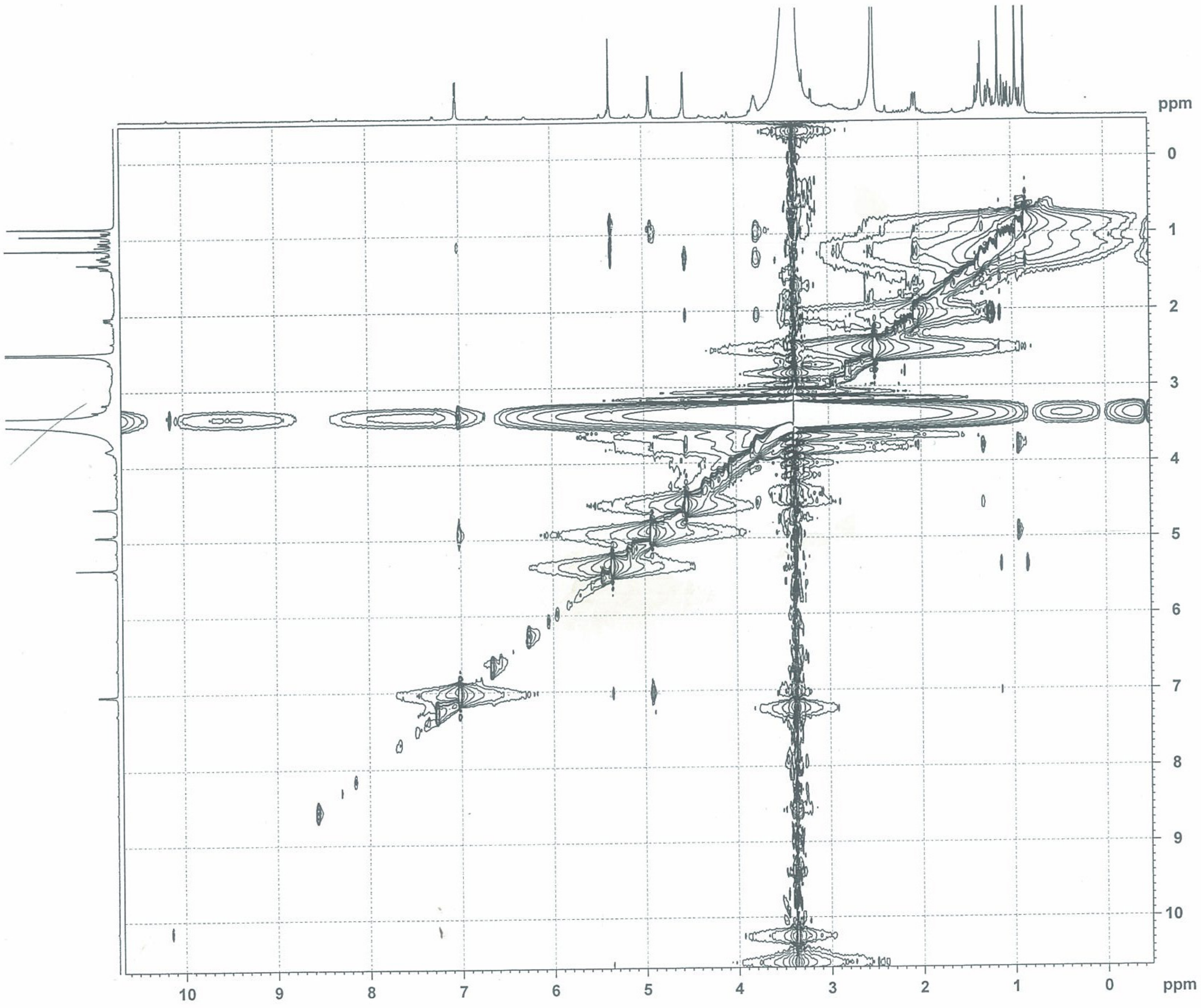

NAME pengyan-S-E-3(1)
EXPNO 7
PROCNO 1
Date_ 20120907
Time 21.49
INSTRUM spect
PROBHD 5 mm PABBO BB-
PULPROG noesyph
TD 2048
SOLVENT DMSO
NS 32
DS 4
SWH 5580.357 Hz
FIDRES 2.724784 Hz
AQ 0.1836404 sec
RG 64
DW 89.600 usec
DE 6.50 usec
TE 297.9 K
D0 0.00007241 sec
D1 1.00000000 sec
D8 0.30000001 sec
IN0 0.00017920 sec

======== CHANNEL f1 ========
NUC1 1H
P1 13.50 usec
PL1 1.00 dB
PL1W 8.77915382 W
SFO1 500.1325507 MHz
ND0 1
TD 128
SFO1 500.1326 MHz
FIDRES 43.596710 Hz
SW 11.158 ppm
FnMODE States-TPPI
SI 1024
SF 500.1300052 MHz
WDW QSINE
SSB 2
LB 0.00 Hz
GB 0
PC 1.00
SI 1024
MC2 States-TPPI
SF 500.1300052 MHz
WDW QSINE
SSB 2
LB 0.00 Hz
GB 0
