# Supplementary figures and images for: Norisoprenoids from the Brown Alga Sargassum naozhouense Tseng et Lu
Source: Molecules. 2018 Feb 7;23(2):348. doi: 10.3390/molecules23020348 (PMC6017521; doi:10.3390/molecules23020348)

Instrument: DSQ (Thermo)  
Ionization Method: EI  
D:\DSQ\DATA-LR\12\102501

10/25/2012 11:51:12 AM

S-E-3(1)

102501 #79 RT: 2.04 AV: 1 NL: 2.68E7  
T: + c Full ms [45.00-800.00]

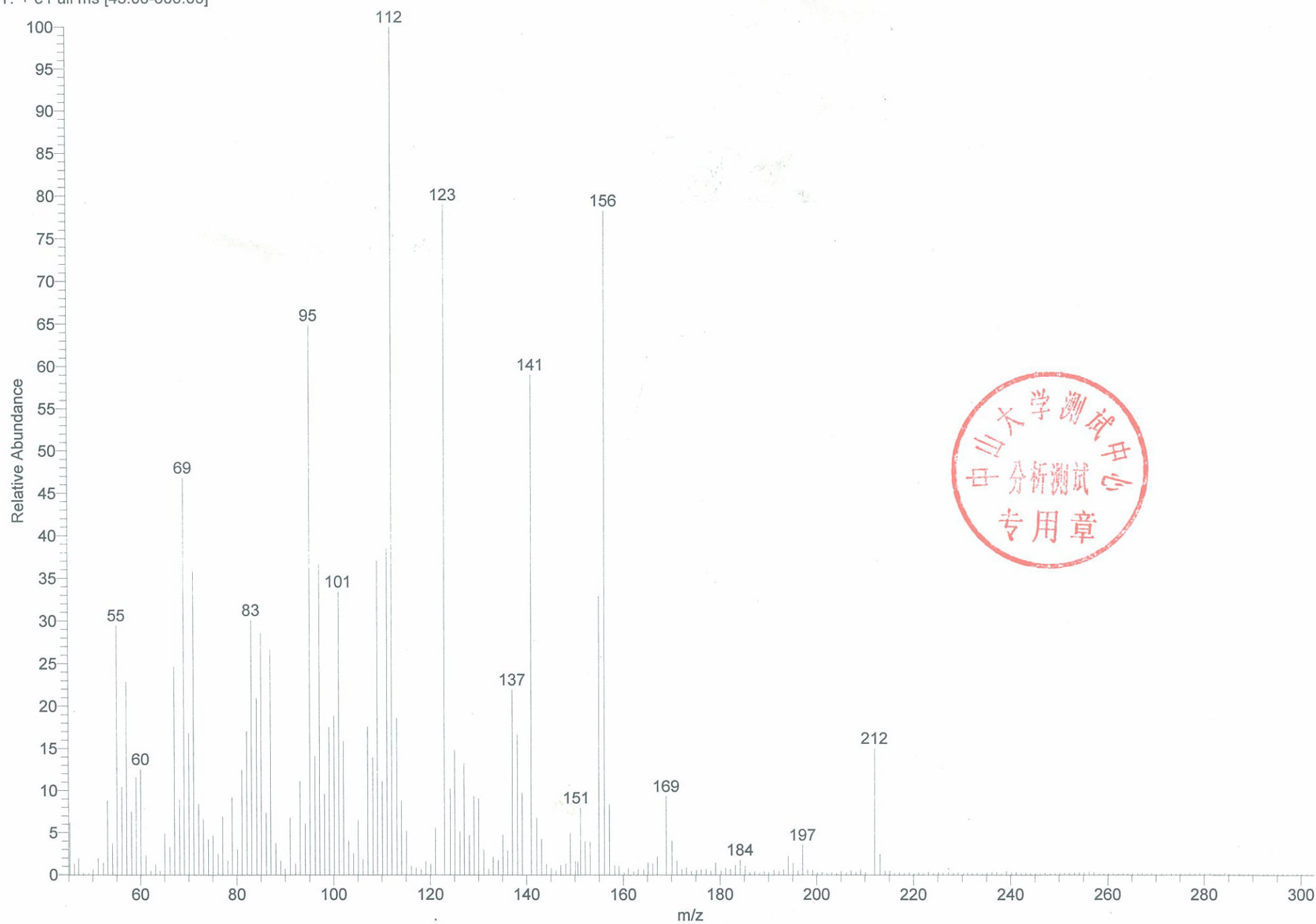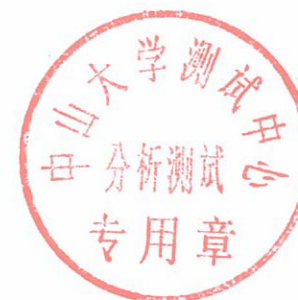

Supplement: Supplementary file 1 [file molecules-23-00348-s001.zip › Supplementary files/1(EI╞╫).pdf]

# **<sup>13</sup>C NMR Spectrum of S-E-3(1)**

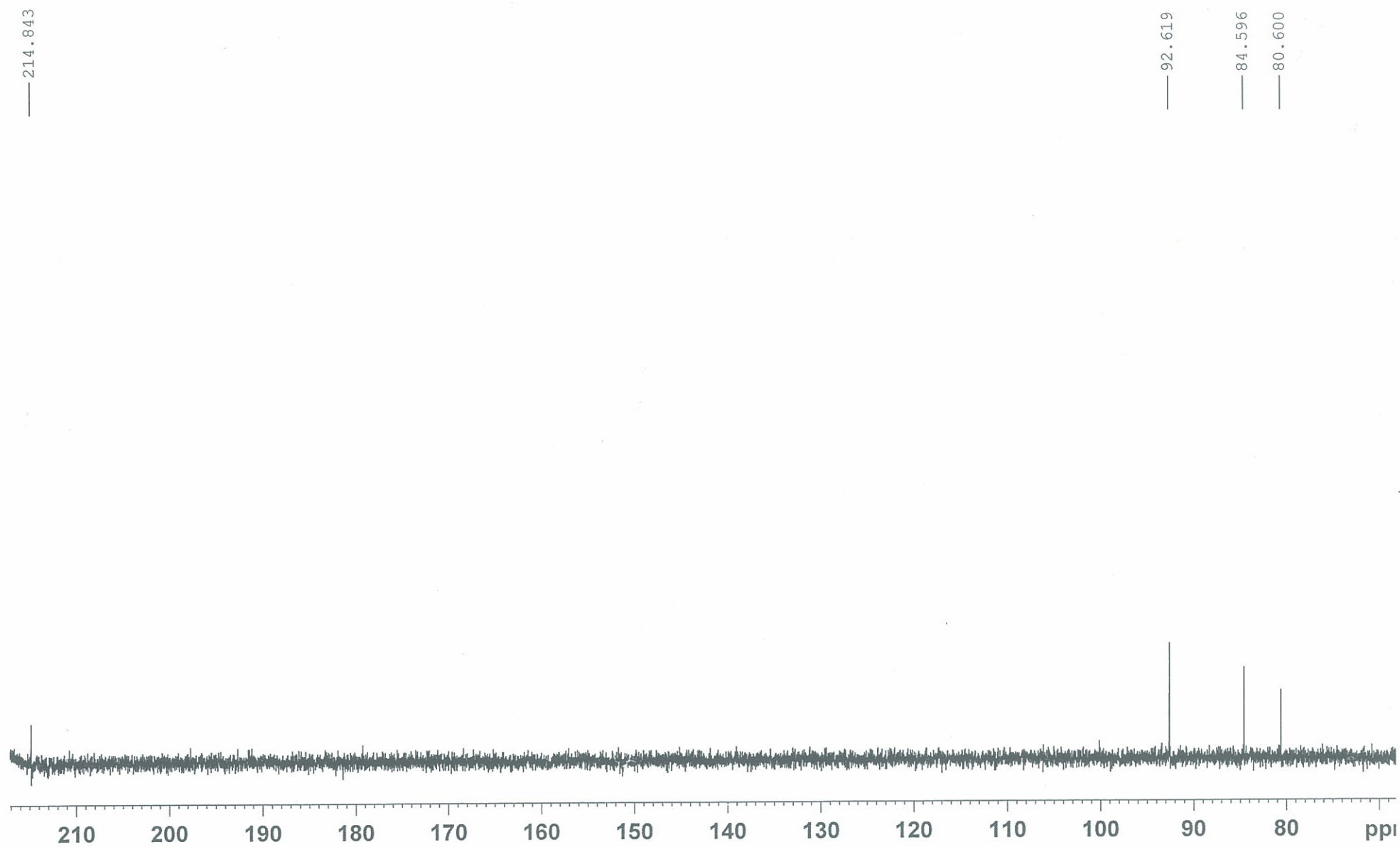

Supplement: Supplementary file 1 [file molecules-23-00348-s001.zip › Supplementary files/3(C╞╫1).pdf]

13C NMR Spectrum of S-E-3(1)

92.62  
84.60  
80.60  
61.61  
44.42  
43.14  
39.93  
39.76  
39.60  
39.43  
39.26  
39.09  
38.93  
37.13  
27.57  
26.07  
25.74

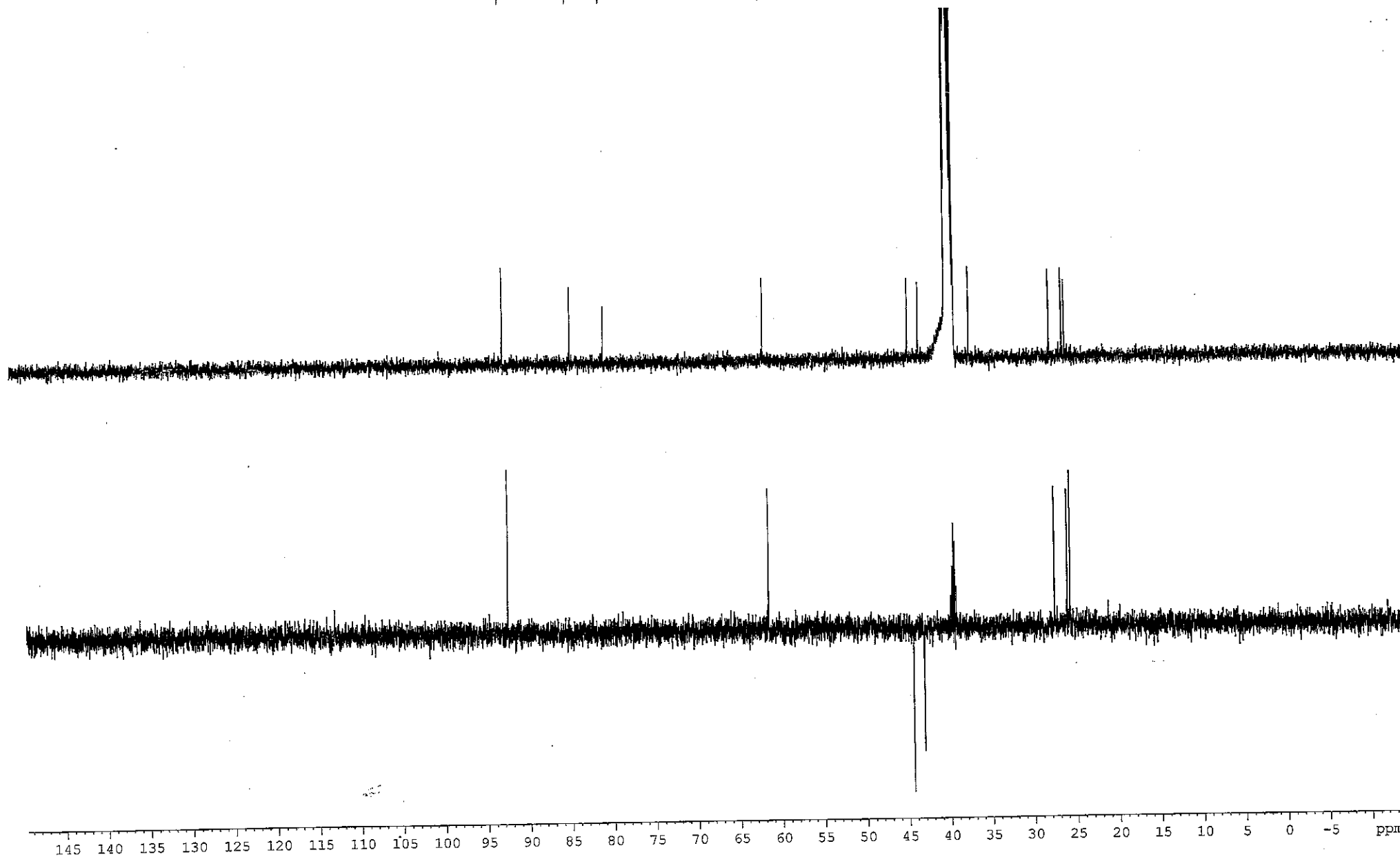

Supplement: Supplementary file 1 [file molecules-23-00348-s001.zip › Supplementary files/5(DEPT).pdf]
